# Supplementary figures and images for: Regnase-1 Deficiency Restrains Klebsiella pneumoniae Infection by Regulation of a Type I Interferon Response
Source: mBio. 2022 Feb 1;13(1):e03792-21. doi: 10.1128/mbio.03792-21 (PMC8805030; doi:10.1128/mbio.03792-21)

Figure S1

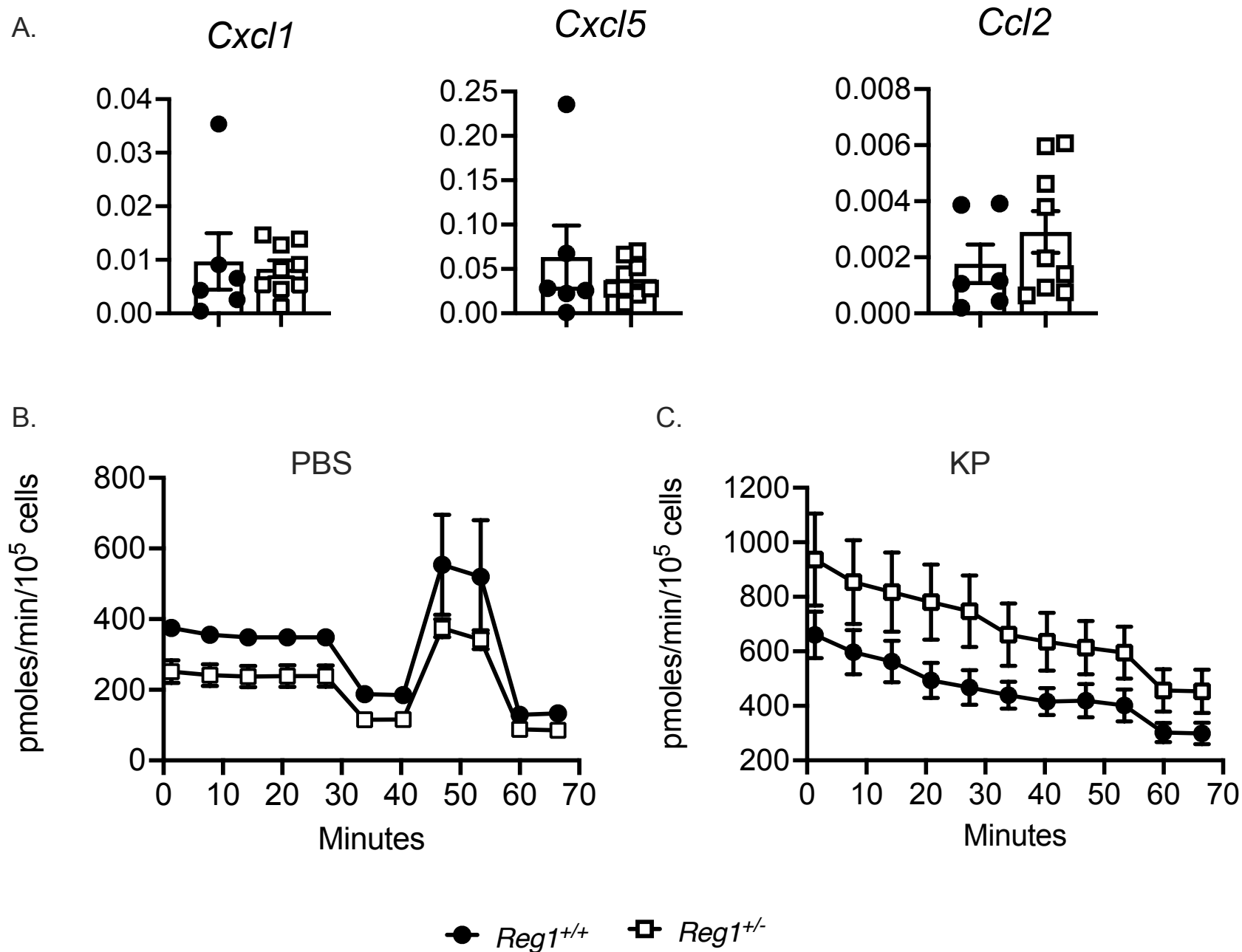

Supplement: FIG S1 [file mbio.03792-21-sf001.pdf]

Figure S2

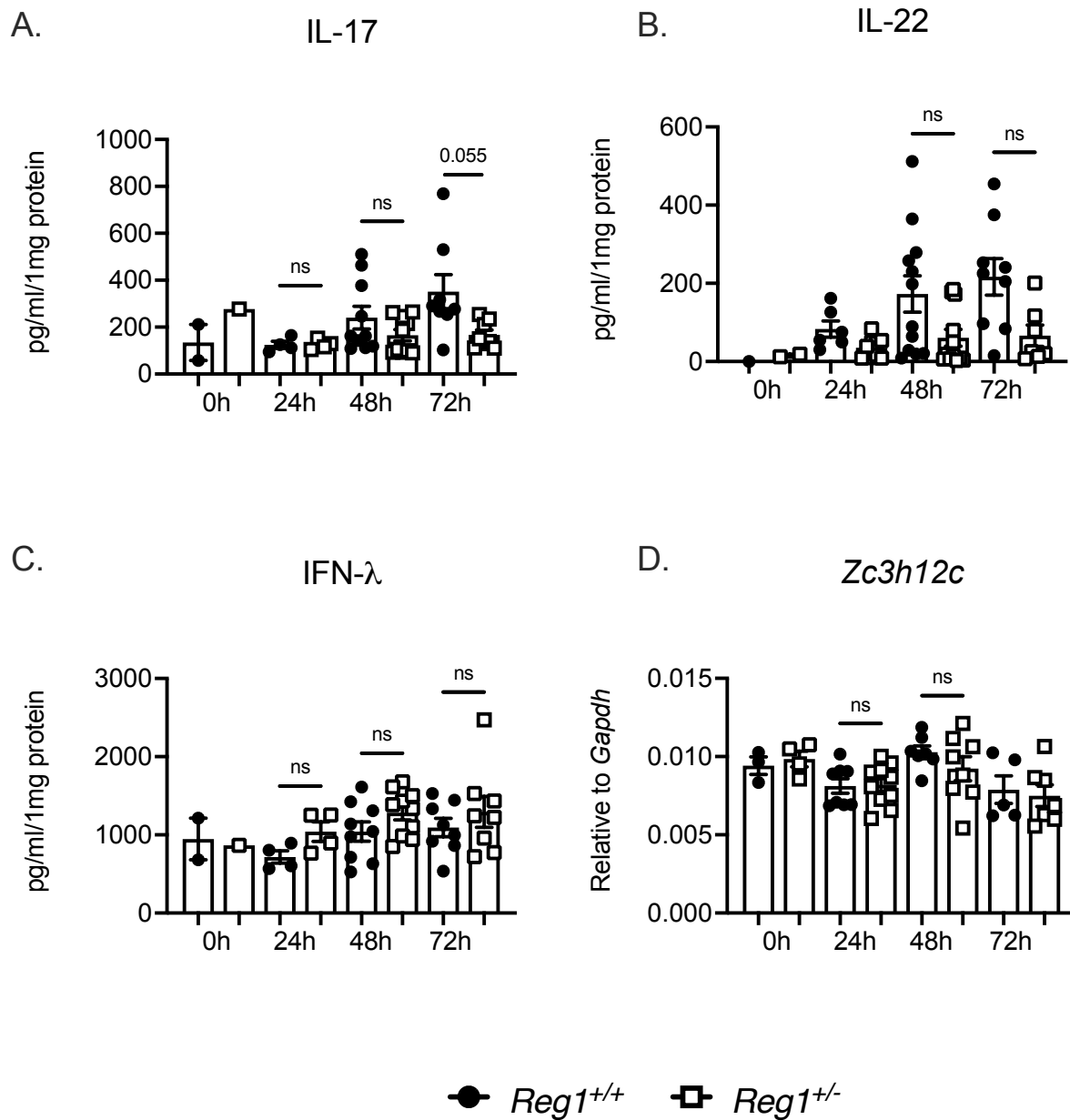

Supplement: FIG S2 [file mbio.03792-21-sf002.pdf]
